# Implementação da Política Nacional de Saúde da Pessoa com Deficiência no Município do Rio de Janeiro, Brasil: avaliação do acesso à saúde bucal

**DOI:** 10.1590/0102-311XPT219725

**Published:** 2026-07-31

**Authors:** Fernanda Nunes Marques Alves, Gisele O’Dwyer

**Affiliations:** 1 Escola Nacional de Saúde Pública Sergio Arouca, Fundação Oswaldo Cruz, Fiocruz, Rio de Janeiro, Brasil.

**Keywords:** Saúde Bucal, Acesso aos Serviços de Saúde, Pessoas com Deficiência, Avaliação em Saúde, Oral Health, Access to Health Services, Persons with Disabilities, Health Evaluation, Salud Bucal, Acceso a los Servicios de Salud, Personas con Discapacidad, Evaluación en Salud

## Abstract

Trata-se de um estudo de caso para avaliar o acesso das pessoas com deficiência (PcD) aos serviços da rede pública de saúde do Município do Rio de Janeiro, Brasil, segundo os níveis de análise: atenção primária à saúde (APS), atenção especializada e atenção hospitalar. O acesso e suas dimensões (acessibilidade, disponibilidade e adequação) foram os critérios de julgamento para aferir o grau de implantação da atenção à saúde bucal no âmbito da Política Nacional de Saúde da Pessoa com Deficiência. Utilizou-se em paralelo as abordagens quantitativa e qualitativa com o emprego de múltiplas fontes e técnicas de coleta de dados. O acesso das PcD aos serviços de saúde bucal foi considerado “incipiente”, sendo os pontos mais críticos da implantação: qualificação profissional, construção compartilhada do cuidado, recursos humanos e materiais, acessibilidade arquitetônica e oferta de serviços, com mais barreiras do que fatores facilitadores. Entre as barreiras: longa distância dos hospitais de referência a determinadas regiões da cidade; baixa qualidade e pouca integração entre modais de transporte público; violência urbana e inadequação das vias públicas; sensação de insegurança do dentista da APS para atender a PcD; poucas oportunidades para qualificação profissional; baixa frequência de apoio matricial dos Centros de Especialidades Odontológicas à APS; baixa cobertura de saúde bucal na APS; oferta limitada de atendimento odontológico sob anestesia geral. A implantação da política vem ocorrendo de forma paulatina sem considerar de forma consistente as necessidades de saúde das PcD. As recomendações deste estudo podem contribuir para o cuidado integral das PcD por meio da qualificação dos componentes da rede no Município do Rio de Janeiro.

## Abstract (en)

This case study evaluated the access of people with disabilities (PwD) to public health network services in Rio de Janeiro, Brazil in the primary health (PHC), specialized, and hospital care levels. Access dimensions (accessibility, availability, and adequacy) constituted the criteria to judge the degree of implementation of oral health care within the Brazilian National Health Policy for Persons with Disabilities. Quantitative and qualitative approaches and multiple sources and data collection techniques were used. This study considered the access of PwD to oral health services as “incipient”, with the most critical points of implementation being professional training, shared construction of care, human and material resources, architectural accessibility, and provision of services. Barriers outnumbered facilitators. The former included long distances from reference hospitals to certain regions of the city, low quality and little integration between public transport modes, urban violence and inadequacy of public roads, insecurity of PHC dentists in assisting PwD, few opportunities for professional training, low frequency of matrix support from the Dental Specialties Centers to PHC, low oral health coverage in PHC, and limited offer of dental care under general anesthesia. The implementation of the policy has been taking place gradually without consistently considering PWD’s health needs. The recommendations in this study can contribute to the comprehensive care of PWD by improving the components of the network in Rio de Janeiro.

## Abstract (es)

Se trata de un estudio de caso para evaluar el acceso de las personas con discapacidad (PcD) a los servicios de salud pública en la ciudad de Río de Janeiro, Brasil, de acuerdo con los niveles de análisis: atención primaria de salud (APS), atención especializada y atención hospitalaria. El acceso y sus dimensiones (accesibilidad, disponibilidad y adecuación) fueron los criterios de evaluación del grado de implementación de la atención de salud bucodental en el ámbito de la Política Nacional de Salud para Personas con Discapacidad. Se utilizaron enfoques cuantitativos y cualitativos en paralelo y múltiples fuentes y técnicas de recopilación de datos. El acceso de las PcD a los servicios de salud bucodental se consideró “incipiente”, y los puntos más críticos de implementación fueron cualificación profesional, construcción compartida de cuidados, recursos humanos y materiales, accesibilidad arquitectónica y prestación de servicios, con más barreras que factores facilitadores. Entre las barreras están larga distancia de los hospitales de referencia a ciertas regiones de la ciudad; baja calidad y poca integración entre los modos de transporte público; violencia urbana e inadecuación de las vías públicas; sensación de inseguridad del odontólogo de APS para atender a las PcD; pocas oportunidades de cualificación profesional; baja frecuencia de apoyo matricial de los Centros de Especialidades Odontológicas a la APS; baja cobertura de salud bucodental en la APS; y limitada oferta de atención odontológica bajo anestesia general. La implementación de la política viene llevándose a cabo gradualmente sin tener en cuenta de manera consistente las necesidades de salud de las PcD. Este estudio puede contribuir a la atención integral de las PcD mediante la cualificación de los componentes de la red en el Municipio de Río de Janeiro.

## Introdução

O acesso das pessoas com deficiência (PcD) aos serviços de saúde é fundamental para promover sua autonomia e inclusão social ^1^, mas ainda enfrenta desafios globais, especialmente em países de baixa e média renda ^2^ ^,^ ^3^, resultando em uma persistente iniquidade em saúde ^4^. Em sistemas universais de saúde, políticas de inclusão que integram a atenção primária à saúde (APS) e a atenção especializada (AE) ajudam a ampliar a cobertura e a continuidade do cuidado, embora persistam problemas relacionados ao acesso ^5^ ^,^ ^6^ ^,^ ^7^ ^,^ ^8^ ^,^ ^9^. É essencial incorporar as necessidades das PcD no planejamento e financiamento dos sistemas de saúde, garantindo políticas públicas inclusivas e eficazes ^10^.

No Brasil, a história recente de formulação e implantação de políticas para atenção à saúde busca a garantia dos direitos fundamentais das PcD de acesso a bens e serviços para todos, com equiparação de oportunidades ^11^. Em 2002, foi instituída a Política Nacional de Saúde da Pessoa com Deficiência (PNSPD) para implementar respostas às complexas questões que envolvem a atenção à saúde das PcD no Brasil e propor estratégias visando à atenção integral à saúde da PcD ^1^. Essas respostas foram materializadas em 2011 no *Plano Nacional dos Direitos da Pessoa com Deficiência - Viver sem Limite* ^12^. No mesmo ano, foi instituída a Rede de Cuidados à Pessoa com Deficiência (RCPD) para ampliar o acesso ao cuidado integral à saúde da PcD ^13^. Em 2023, foi instituído o Novo Viver sem Limite ^14^ e houve atualizações da PNSPD ^15^ buscando promover e proteger a saúde da PcD por meio da ampliação do acesso ao cuidado integral no âmbito do Sistema Único de Saúde (SUS), em articulação com as demais políticas e ações intersetoriais.

A PNSPD se articula com a Política Nacional de Saúde Bucal (PNSB), cabendo à APS organizar o acesso a ações para atendimento e referência de casos complexos para a AE e a atenção hospitalar (AH), especialmente dos pacientes com necessidades especiais (PNE) que não podem receber atendimento odontológico convencional ^16^. Na AE, os Centros de Especialidades Odontológicas (CEO) devem fortalecer, ampliar e qualificar os cuidados às PcD que necessitem de atendimento no âmbito das especialidades odontológicas ^16^, com incentivos financeiros adicionais para os CEO que fizerem parte da RCPD mediante cumprimento de compromissos mínimos estabelecidos pelo Ministério da Saúde ^17^. Na AH, é prevista a ampliação do acesso às urgências e emergências odontológicas, bem como o atendimento sob sedação ou anestesia geral das PcD, adequando centros cirúrgicos e equipes para este fim ^13^.

Passados mais de 20 anos após a instituição da PNSPD, é essencial analisar o seu processo de implantação, levando em consideração as particularidades das redes de atenção à saúde (RAS) em diferentes contextos e territórios para acompanhar e verificar em que medida a política tem contribuído para o cumprimento dos princípios e das diretrizes do SUS. Diante dos desafios para a atenção à saúde das PcD nas RAS, a PNSPD destaca a importância de acompanhamento e avaliação contínuos para o aprimoramento de suas ações de modo a embasar decisões, justificar escolhas e aumentar a eficácia dos programas e políticas, com vistas à melhoria social ^18^ ^,^ ^19^. Considerando que a avaliação de implantação do tipo 1b visa a explicar a diferença entre a intervenção tal como foi planejada e aquela realmente implantada, ela ajuda a compreender as variações observadas no grau e no nível de integralidade de sua implantação ^18^.

O Município do Rio de Janeiro, de acordo com *Censo Demográfico* de 2022 ^20^, tinha uma população estimada de 6.211.223 habitantes, sendo que 21,73% residiam em favelas e 6,9% tinham algum tipo de deficiência. Dado que uma parte significativa das PcD reside em áreas de difícil acessibilidade, torna-se fundamental refletir sobre os direitos e as desigualdades no acesso aos serviços de saúde ^21^. Ademais, mudanças na Política Nacional de Atenção Básica (PNAB) trouxeram descontinuidades em aspectos essenciais da APS ^22^, incluindo a extinção de 55 equipes de saúde bucal (eSB) entre 2016 e 2018, o que reduziu a cobertura de 32% para 27,1% ^23^.

Este estudo teve como objetivo avaliar o acesso das PcD aos serviços odontológicos da rede pública de saúde do Município no Rio de Janeiro pela aferição do grau de implantação no âmbito da PNSPD.

## Materiais e métodos

Trata-se de uma pesquisa de avaliação de implantação do tipo 1b, desenvolvida entre 2022 e 2023, que adotou como estratégia metodológica o estudo de caso do tipo único (rede pública de saúde bucal do Município do Rio de Janeiro) com níveis de análise imbricados (APS, AE e AH). A avaliação foi desenvolvida como etapa metodológica da tese de doutorado de Alves ^24^, sendo conduzida após Estudo de Avaliabilidade.

No município, o programa de saúde bucal está organizado em uma rede de serviços integrada, regionalizada e hierarquizada, onde o cuidado aos usuários é realizado em diferentes níveis de atenção: atenção primária (centros municipais de saúde e clínicas da família); atenção secundária (CEO); atenção terciária (unidades de pronto atendimento, hospitais gerais e especializados) ^25^. O município é bastante heterogêneo e apresenta diferentes graus de desenvolvimento e desigualdade na distribuição e utilização dos serviços de saúde ^26^. Está inserido na Região Metropolitana de Saúde I, com a divisão em dez Áreas de Planejamento (AP): 1.0 (região do Centro e zona portuária); 2.1 (zona sul); 2.2, 3.1, 3.2 e 3.3 (zonas norte e Leopoldina); 4.0, 5.1, 5.2 e 5.3 (zona oeste) ^26^.

Para avaliação da implantação da PNSPD, o acesso e suas dimensões foram tomados como critérios. Considerando seu caráter multidimensional, o embasamento teórico do estudo se apoiou em Andersen ^27^ que considera o acesso como a entrada no serviço de saúde e a continuidade do tratamento; no modelo sistêmico de Donabedian ^28^ que observa a relação entre os componentes da estrutura, do processo e do resultado; e nas dimensões acessibilidade, adequação e disponibilidade, adotadas por Penchansky & Thomas ^29^, que são imbricadas e complementares, na medida em que representam pontos essenciais que remetem ao sentido da integralidade da atenção na RAS.

**Figura 1:**
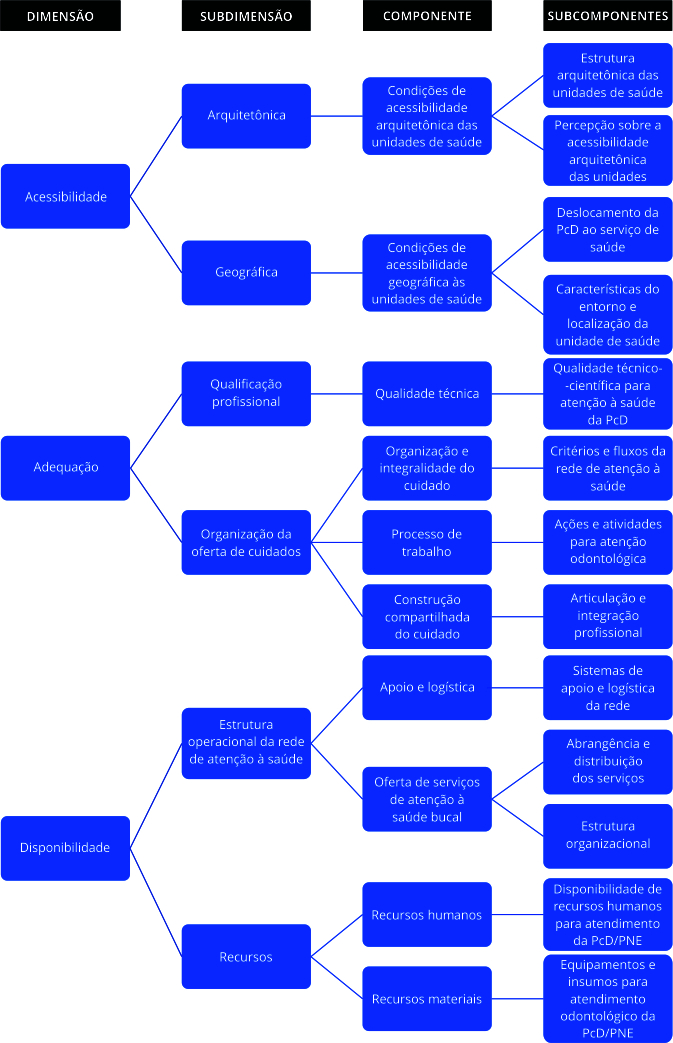
Modelo teórico de avaliação do acesso das pessoas com deficiência (PcD) aos serviços de atenção à saúde bucal da rede pública no Município do Rio de Janeiro, Brasil, 2022.

Com base nas dimensões do acesso, suas subdimensões e seus respectivos componentes, subcomponentes e critérios/indicadores, foi estruturado o modelo teórico da avaliação (Figura 1), que possibilitou a construção da matriz de análise e julgamento do acesso das PcD aos serviços odontológicos (Quadro 1), estruturada com critérios e indicadores definidos e organizados para cada dimensão/subdimensão.

PNE: paciente com necessidades especiais. Fonte: elaboração própria.

**Quadro 1:** Matriz de análise e julgamento do acesso da pessoa com deficiência (PcD) aos serviços de saúde bucal da rede pública do Município do Rio de Janeiro, Brasil.

SUBCOMPONENTE	CRITÉRIOS/INDICADORES	COMO FOI MEDIDO	FONTES DE VERIFICAÇÃO
ACESSIBILIDADE			
Percepção sobre a acessibilidade arquitetônica das unidades	Existência de critérios da acessibilidade nas unidades de saúde: rampas de acesso, corrimão em rampas, elevadores (em caso de degraus/escadas para acesso aos andares superiores), vagas de estacionamento para PcD, corredores e portas largas que permitam a passagem de cadeira de rodas, informação em Braille, sala de espera adequada com sanitário acessível, cadeira de rodas em condições de uso, disponível para deslocamento do usuário, banheiro adaptado e piso tátil, ausência de degraus/escadas para acesso ao atendimento	Proporção de CDAB que apontaram a existência de cada critério de acessibilidade das PcD nas unidades de APS	Questionário dos CDAB
		Proporção de gestores de CEO que apontaram a existência de cada critério de acessibilidade das PcD nos CEO	Questionário dos gestores dos CEO
		Média da existência de cada critério de acessibilidade nas unidades de AH visitadas	Observação direta dos serviços de AH
	UBS com acessibilidade e mobilidade para PcD	Proporção de CDAB que consideraram que as instalações da UBS foram projetadas de forma conveniente para acesso da PcD	Questionário dos CDAB
	CEO com acessibilidade e mobilidade para PcD	Proporção de gestores de CEO que consideraram que as instalações da UBS foram projetadas de forma conveniente para acesso da PcD	Questionário dos gestores dos CEO
	Classificação de acessibilidade arquitetônica da UBS, segundo CDAB	Média dos valores atribuídos à percepção dos CDAB sobre as condições de acessibilidade relacionadas à estrutura física das UBS	Questionário dos CDAB
	Classificação de acessibilidade arquitetônica do CEO, segundo chefe CEO	Proporção de gestores dos CEO que consideraram que as instalações do CEO proporcionam acessibilidade total às PcD	Questionário dos gestores dos CEO
	Classificação de acessibilidade arquitetônica da unidade de AH, segundo acompanhante da PcD/PNE	Média dos valores atribuídos à percepção dos acompanhantes sobre as condições de acessibilidade relacionadas à estrutura física dos hospitais em que PNE são atendidos	Questionário dos acompanhantes dos usuários
Deslocamento da PcD ao serviço de saúde	Distância percorrida, tempo gasto e custo indireto para deslocamento da PcD entre seu domicílio e a unidade de AE e de AH utilizando transporte público	Média da distância percorrida (em km), do tempo (em minutos) e do custo indireto (quantidade de meio de transporte utilizado) para deslocamento da PcD entre seu domicílio e o CEO/unidade de AH utilizando transporte público	Banco do SISREG
	Deslocamento da PcD/PNE até a UBS e unidades que oferecem atenção odontológica especializada (CEO e hospitais)	Média do grau de dificuldade/facilidade para deslocamento da PcD/PNE até a UBS e unidades que ofertam atenção odontológica especializada, segundo CDAB	Questionários dos CDAB
Características do entorno e localização da unidade de saúde	Existência/ausência de barreiras de acesso entre o domicílio/território e o hospital	Proporção de unidades de AH visitadas que apresentam em seu entorno: via pavimentada, transporte público, calçadas adequadas para o deslocamento das PcD	Observação direta dos serviços de AH
			Questionários dos acompanhantes dos usuários
	Existência/ausência de barreiras de acesso entre o domicílio/território e UBS	Proporção de CDAB que apontaram a ausência/existência de terreno acidentado/íngreme/com encostas/inclinações; terreno irregular/sem pavimentação; escadas; semáforos com faixa de pedestres no trajeto do paciente entre o domicílio/território e a UBS; e de rampas de acesso em calçadas no trajeto do paciente entre o domicílio/território às UBS	Questionários dos CDAB
	Localização conveniente para as PcD chegarem de transporte público ou particular à unidade de saúde	Proporção de CDAB que consideraram a localização da UBS conveniente para as PcD chegarem usando transporte público ou particular	Questionários dos CDAB
		Proporção de gestor de CEO que consideraram a localização do CEO conveniente para as PcD chegarem usando transporte público ou particular	Questionários dos gestores dos CEO
		Média da percepção dos acompanhantes da PcD/PNE quanto à localização da unidade AH	Questionários dos acompanhantes dos usuários
ADEQUAÇÃO			
Qualidade técnico científica para atenção à saúde da PcD	Segurança do CDAB para realizar o atendimento odontológico do PNE	Proporção de CDAB que: se sentem seguros para realizar o atendimento odontológico do PNE	Questionários dos CDAB
	Educação continuada para CDAB sobre atendimento odontológico do PNE	Proporção de CDAB que participaram de cursos ou treinamentos sobre atendimento odontológico do PNE durante o tempo de atuação da rede municipal	
	Formação do CDAB para atendimento odontológico à PcD/PNE	Proporção de CDAB que cursaram disciplina na graduação que tratasse do atendimento odontológico à PcD/PNE	
	Educação continuada para CD, ASB e TSB da AE sobre atendimento odontológico do PNE	Proporção de CD do CEO, de ASB e TSB que participaram de cursos ou treinamentos para atendimento odontológico à PcD/PNE nos últimos 5 anos	Questionários dos gestores dos CEO
	Formação do CD do CEO que atua na especialidade de “Odontologia para PNE”	Proporção de CD do CEO que atuam na especialidade de “Odontologia para PNE” com especialização na área	
	Formação do CDAH que atua na especialidade de “Odontologia para PNE”	Proporção de CDAH que atuam na especialidade de “Odontologia para PNE” com especialização na área	Entrevistas com responsáveis dos serviços de Odontologia da AH e CDAH
Critérios e fluxos da rede de atenção à saúde	Existência de protocolo de critérios e fluxos para a atenção odontológica da PcD/PNE nos serviços da rede municipal	Verificação da existência de protocolo de critérios e fluxos para a atenção odontológica da PcD/PNE nos serviços da rede municipal	Entrevista com coordenador de saúde bucal
	Frequência com que contrarreferência dos CEO e dos hospitais chega aos serviços de APS	Média de frequência com que contrarreferência dos CEO e dos hospitais chega aos serviços de APS	Questionários dos CDAB
	Conhecimento do protocolo, dos critérios e dos fluxos para a atenção odontológica da PcD/PNE nos serviços da rede municipal pelos CDAB	Proporção de CDAB que conhecem o protocolo, os critérios e os fluxos para a atenção odontológica da PcD/PNE	
	Conhecimento sobre o PADI pelo CDAB	Proporção de CDAB que conhecem o PADI	
	Resposta do CEO sobre o atendimento odontológico do PNE por meio de contrarreferência ao serviço da APS	Proporção de CEO que buscam garantir resposta sobre o atendimento odontológico do PNE por meio de contrarreferência ao serviço da APS	Questionários dos gestores dos CEO
	Resposta da AH sobre o atendimento odontológico do PNE por meio de contrarreferência ao serviço da APS	Média da frequência com que os entrevistados da AH respondem ao CDAB por meio de contrarreferência	Entrevistas com responsáveis dos serviços de Odontologia da AH e CDAH
Ações e atividades para a atenção odontológica	Identificação das PcD da área de abrangência da UBS pelo CDAB	Média do grau de identificação das PcD da área de abrangência da UBS pelo CDAB	Questionários dos CDAB
	Conhecimento pelo CDAB sobre: o perfil das PcD que residem na área de abrangência da ESF (quantidade, tipos de deficiência possuem, quais PcD demandam cuidado odontológico especializado); como consultar informações no sistema de informação/prontuário eletrônico sobre o perfil das PcD	Proporção de CDAB que: conhecem o perfil das PcD da área de abrangência da UBS; sabem consultar informações sobre o perfil das PcD no sistema de informação; realizam levantamento das condições de saúde bucal das PcD que demandam cuidado odontológico especializado; realizam busca ativa para levantar as condições de saúde bucal das PcD/PNE	
	Levantamento das condições de saúde bucal das PcD que demandam cuidados especiais em Odontologia da área de abrangência de sua unidade de saúde/equipe de saúde da família		
	Busca ativa das PcD que demandam cuidados especiais em Odontologia da área de abrangência de sua unidade/equipe de saúde da família		
	Acolhimento/modo de recepção realizado pela equipe ao PNE, segundo CDAB e gestores do CEO	Médias dos valores das respostas quanto à avaliação atribuída pelos CDAB ao acolhimento da PcD/PNE na UBS	
	Prioridade de agendamento/atendimento odontológico da PcD na APS	Proporção de CDAB que priorizam o agendamento/atendimento da PcD	
	Acompanhamento do PNE após alta do atendimento especializado	Média da frequência com que o CDAB consegue fazer o acompanhamento do PNE após alta do atendimento especializado	
	Acolhimento/modo de recepção realizado pela equipe ao PNE no CEO	Médias dos valores das respostas quanto à avaliação atribuída pelos gestores dos CEO ao acolhimento da PcD/PNE no CEO	Questionários dos gestores dos CEO
	Manutenção da produção mensal mínima das atividades no CEO	Proporção de CEO que: mantém a produção mensal mínima; alimentam regularmente no SIA-SUS; informam no SCNES - Serviço de Atenção em Saúde Bucal, classificação 007 - Atendimento à PcD pelo CEO; disponibilizam 40 horas semanais, no mínimo, para atendimento exclusivo à PcD; realizam ações de promoção da saúde	
	Alimentação regular e consistente da informação de produção procedimentos realizados em PcD pelo CEO		
	Tempo para atendimento exclusivo à PcD no CEO		
	Realização de ações de promoção da saúde nos CEO		
	Sistema de agendamento da PcD/PNE nas unidades de AH visitadas	Média dos valores atribuídos pela pesquisadora ao sistema de agendamento da PcD/PNE nas unidades de AH visitadas	Observação direta dos serviços de AH
	Acolhimento/modo de recepção à PcD/PNE no CEO e na AH	Média dos valores atribuídos pelos entrevistados da AH à recepção/acolhimento da PcD/PNE nas unidades de AH visitadas	Entrevistas com responsáveis dos serviços de Odontologia da AH e CDAH
	Realização de procedimentos odontológicos em caráter de urgência e sob sedação/anestesia geral nas unidades de AH em quantidade suficiente frente à demanda de atendimento das PcD/PNE	Proporção de unidades de AH visitadas que realizam procedimentos odontológicos sob sedação/anestesia geral; atendimento odontológico em caráter de urgência/emergência às PcD/PNE	
Articulação e integração profissional	Integração do trabalho da saúde bucal com profissionais de outras categorias para programar ações e definir prioridades de atuação junto ao PNE e suas famílias/cuidadores	Média dos valores das respostas relacionadas à percepção dos CDAB quanto à integração do trabalho da saúde bucal com a equipe de saúde da família ou com profissionais de outras categorias para programar ações e definir prioridades de atuação junto ao PNE e suas famílias/cuidadores	Questionários dos CDAB
	Suporte dos profissionais da eMulti e do CEO ao atendimento/acompanhamento do PNE	Frequência com que a equipe eMulti realiza matriciamento para suporte ao atendimento da PcD/PNE, segundo o CDAB	
		Frequência com que os CDAB que recebem apoio de profissionais do CEO para o atendimento/acompanhamento do PNE	
	Apoio de profissionais do PADI na atenção em saúde bucal do PNE no território de sua atuação da APS	Frequência com que os CDAB que recebem apoio de profissionais do PADI na atenção em saúde bucal da PcD que possuem doenças crônico-degenerativas com restrição/impedimento de locomoção realizado em domicílio no território de sua atuação da APS	
	CEO como referência para a APS	Proporção de CEO que são referência para profissionais/eSB da atenção básica de sua área de abrangência	Questionários dos gestores dos CEO
	Matriciamento realizado pelos profissionais do CEO junto aos profissionais/eSB da APS	Proporção de CEO que realizam matriciamento às eSB da APS para atenção à saúde bucal da PcD/PNE	
	Discussão de casos clínicos de PcD/PNE difíceis e desafiadores, atividades clínicas compartilhadas, visitas domiciliares, construção conjunta de projetos terapêuticos para a PcD/PNE, atividades de educação permanente, construção e discussão de protocolos clínicos, pactuação de critérios para referência da especialidade de Odontologia para PNE e interconsulta sobre atenção à saúde da PcD/PNE conjuntas realizadas entre os profissionais do CEO e equipes da APS	Proporção de CEO que realizam: discussão de casos; atividades clínicas; visitas domiciliares; construção conjunta de projetos terapêuticos para a PcD/PNE; atividades de educação permanente; construção e discussão de protocolos clínicos; pactuação de critérios para referência da especialidade de Odontologia para PNE; interconsulta para resolução de casos considerados complexos junto aos profissionais/eSB da APS relacionadas à atenção à saúde bucal da PcD/PNE	
	Relação entre eSB da AH com os demais profissionais/equipes da AH para construção compartilhada de plano terapêutico do PNE	Média da classificação atribuída pelos profissionais entrevistados sobre a relação entre eSB da AH com os demais profissionais/equipes da AH para construção compartilhada de plano terapêutico do PNE	Entrevistas com responsáveis dos serviços de Odontologia da AH e CDAH
DISPONIBILIDADE			
Sistemas de apoio e logística da rede	Sistema de prontuário eletrônico em saúde na APS, AE e AH	Proporção de CDAB, de CEO e de CDAH que dispõem de prontuário eletrônico para registro de informações e atendimentos	Questionários dos CDAB e dos gestores dos CEO
			Entrevistas com os responsáveis dos serviços de Odontologia da AH e CDAH
			Observação direta dos serviços de AH
	Transporte sanitário para PcD/PNE para procedimentos eletivos de saúde bucal	Proporção de CDAB que dispõe de transporte sanitário para PNE para atendimento eletivo especializado de saúde bucal	Questionários dos CDAB
	Exames pré-operatórios na AH	Proporção de unidades de AH que ofertam exames pré-operatórios para o PNE	Entrevistas com os responsáveis dos serviços de Odontologia da AH e CDAH
	Sistema de regulação para encaminhamento das PcD/PNE aos serviços odontológicos especializados	Existência de sistema de regulação para encaminhamento dos PNE aos serviços especializados e de central de regulação para agendamento dos atendimentos especializados em Odontologia	Entrevistas com coordenador de saúde bucal
	Central de regulação		
Abrangência e distribuição dos serviços	Cobertura de saúde bucal na atenção básica no município	População cadastrada pelas equipes de Saúde da Família e de APS vinculadas a eSB financiadas pelo Ministério da Saúde no município/estimativa populacional do Município do Rio de Janeiro	e-Gestor Atenção Básica
	Cobertura populacional dos CEO	Número de CEO/estimativa populacional do Município do Rio de Janeiro x 100	Entrevistas com coordenador de saúde bucal
	Especialidade PNE no CEO	Porcentual de CEO que oferece atendimento de especialidade “Odontologia para PNE”	Questionários dos gestores dos CEO
	Adesão dos CEO à RCPD	Porcentual de CEO com adesão à RCPD	
	Hospitais com centro cirúrgico equipado para atendimento odontológico da PcD	Porcentual de unidades de AH visitadas com centro cirúrgico equipado para atendimento odontológico da PcD	Observação direta dos serviços de AH
Estrutura organizacional	Funcionamento das unidades de APS	Proporção de CDAB que informaram sobre funcionamento da unidade com carga horária mínima de 40 horas semanais, no mínimo 5 dias da semana e nos 12 meses do ano	Questionários dos CDAB
	Tempo para atendimento exclusivo à PcD no CEO	Proporção de CEO que disponibilizam 40 horas semanais, no mínimo, para atendimento exclusivo à PcD	Questionários dos gestores dos CEO
	Tempo para agendamento de consulta especializada para “Odontologia para PNE” nos CEO e na AH	Média do tempo (em dias) para agendamento de consulta para “Odontologia para PNE” no CEO e na AH	Banco SISREG
	Funcionamento do serviço de Odontologia ambulatorial e do centro cirúrgico nas unidades de AH visitadas	Porcentual de unidades de AH visitadas que oferecem atendimento odontológico ambulatorial e no centro cirúrgico para PcD/PNE 40 horas semanais	Entrevistas com chefes e CDAH
			Observação direta dos serviços de AH
Recursos humanos para atendimento da PcD/PNE	Quantidade de CDAB em relação à população do município	Razão entre população com cobertura de equipe de APS e de Saúde da Família residente do Município do Rio de Janeiro e o número de dentistas da APS	SCNES
	Quantidade total de CD no CEO	Proporção de CEO que têm quantidade mínima exigida de CD, acordo com o tipo de CEO	Questionários dos gestores dos CEO
	Quantidade de CD no CEO para atendimento do PNE	Proporção de CEO que têm quantidade suficiente de CD para atendimento do PNE	
	Quantidade de ASB e TSB no CEO	Proporção de CEO que têm quantidade suficiente de ASB/TSB para atender as necessidades do serviço	
Recursos humanos para atendimento da PcD/PNE	Quantidade suficiente de CD, ASB, TSB e de médicos anestesistas na AH para atendimento odontológico do PNE	Proporção de unidades de AH que têm CD, ASB e TSB e médicos anestesistas em quantidade suficiente para atendimento odontológico do PNE	Entrevistas com responsáveis dos serviços de Odontologia da AH e CDAH
			Observação direta dos serviços de AH
Insumos, instrumentais e equipamentos para atendimento odontológico da PcD/PNE	Disponibilidade de insumos, instrumentais e equipamentos gerais para atendimento odontológico da PcD/PNE	Média da frequência com que se dispõe de escova de dente, creme dental, fio dental, material de educação em saúde, materiais dentários e cadeira/equipamentos odontológicos em condições de uso na UBS	Questionários dos CDAB
		Proporção de CEO que dispõem de consultórios odontológicos completos, aparelho de RX dentário, caneta de alta e baixa rotação, amalgamador, fotopolimerizador, compressor, instrumentais e materiais odontológicos em quantidade suficiente e em condições de uso	Questionários dos gestores dos CEO
		Proporção de unidades de AH visitadas que dispõem de consultórios odontológicos completos, aparelho de RX dentário, caneta de alta e baixa rotação, amalgamador, fotopolimerizador, aparelho de ultrassom/profilaxia dentária, instrumentais e materiais odontológicos em quantidade suficiente e em condições de uso	Entrevistas com responsáveis dos serviços de Odontologia da AH e CDAH
			Observação direta dos serviços de AH
	Disponibilidade de insumos, instrumentais e equipamentos específicos para atendimento odontológico da PcD/PNE	Proporção de CEO que dispõem de aparelho de sedação consciente, materiais para auxiliar na estabilização protetora no posicionamento do paciente na cadeira odontológica (apoios, almofadas)	Questionários dos gestores dos CEO
		Proporção de unidades de AH visitadas com centro cirúrgico equipado para atendimento odontológico da PcD, aparelho de sedação consciente e de materiais para auxiliar na estabilização protetora do paciente	Entrevistas com responsáveis dos serviços de Odontologia da AH e CDAH
			Observação direta dos serviços de AH

AE: atenção especializada; AH: atenção hospitalar; APS: atenção primária à saúde; ASB: auxiliar de saúde bucal; CD: cirurgião-dentista; CDAB: cirugião-dentista da atenção primária à saúde; CDAH: cirugião-dentista da atenção hospitalar; CEO: Centros de Especialidades Odontológicas; eMulti: equipes multiprofissionais na atenção primária à saúde; eSB: equipes de saúde bucal; ESF: Estratégia Saúde da Família; PADI: Programa de Atenção Domiciliar; PNE: paciente com necessidades especiais; RCPD: Rede de Cuidados à Pessoa com Deficiência; RX: raio X; SCNES: Sistema de Cadastro dos Estabelecimentos de Saúde; SIA-SUS: Sistema de Informação Ambulatorial do Sistema Único de Saúde; SISREG: Sistema Nacional de Regulação; TSB: técnico de saúde bucal; UBS: unidade básica de saúde.

Fonte: elaboração própria.

Utilizou-se em paralelo as abordagens quantitativa e qualitativa, próprias dos métodos integrados ou mistos ^30^. Os dados, advindos de diferentes fontes e técnicas, foram triangulados (no sentido de observar a convergência) e também houve complementação das informações, explicitando as facetas do acesso. Como fontes de verificação, foram levantadas as informações de fonte primária (entrevista e observação participante) e secundária (Sistema Nacional de Regulação, Cadastro Nacional dos Estabelecimentos de Saúde do Brasil - CNES, e documentos).

Foram convidados a participar da pesquisa: o coordenador municipal de saúde bucal (GC); todos os cirurgiões-dentistas (CD) da APS (CDAB), gestores dos 19 CEO e 2 responsáveis pelos serviços de Odontologia (GL) de hospitais municipais de referência em atendimento em “Odontologia para PNE” (H1 e H2). Além destes, participaram 4 CD da AH (CDAH) e acompanhantes de usuários (A) atendidos nestes serviços. Durante o desenvolvimento do trabalho de campo, surgiu a necessidade de entrevistar um CD que atuasse na especialidade Odontologia para PNE (CDAE), com especialização nesta área e prática em sedação, para compreender a oferta de atendimento odontológico ambulatorial sob sedação da PcD no CEO. Um código numerado específico foi atribuído a cada participante para identificar a qual grupo pertencia, mantendo o anonimato de seus dados na apresentação e discussão dos resultados da pesquisa.

No total, participaram: o coordenador de saúde bucal; 17 gestores de CEO e 2 responsáveis pelos serviços de Odontologia da AH, 1 CD do CEO, 4 CDAH e 21 acompanhantes dos usuários da AH. Em relação aos CDAB, em função do número de eSB cadastradas no 1º semestre de 2022 (n = 427), foi calculado por AP a taxa de resposta desejada dos questionários enviados aos CDAB. Mesmo com a ampla divulgação da pesquisa e inúmeras tentativas de contato, participaram 139 CD, o que correspondeu à taxa de resposta de 67,5% (86,7% na AP 1.0, 100% na AP 2.1, 100% na AP 2.2, 100% na AP 3.1, 100% na AP 3.2, 33,3% na AP 3.3, 22,2% na AP 4.0, 52% na AP 5.1, 46,1% na AP 5.2 e 33,3% na AP 5).

Para o levantamento de dados, foram utilizados instrumentos de coleta de dados (questionários e roteiros de entrevistas semiestruturados), com conteúdo específico conforme o grupo de participantes (Quadro 2).

**Quadro 2:** Conteúdo dos instrumentos de coleta de dados primários, segundo grupo de participantes.

GRUPO DE PARTICIPANTES	INSTRUMENTO DE COLETA DE DADOS	CONTEÚDOS ABORDADOS
Coordenador de saúde bucal	Roteiro de entrevista semiestruturado	Educação permanente para os trabalhadores Qualificação do cuidado nos níveis de atenção; planejamento para a construção da RAS Monitoramento e avaliação dos resultados Sistemas de informação Regulação Práticas de trabalho em rede para a atenção à saúde da PcD Desafios da implantação da Política Nacional de Saúde da Pessoa com Deficiência Fatores facilitadores e barreiras de acesso das PcD aos serviços da rede pública municipal
CDAB	Questionário	Acessibilidade geográfica Estrutura arquitetônica da UBS e dos consultórios odontológicos Qualificação profissional (cursos/treinamentos e segurança do CDAB para realizar atendimento da PcD Comunicação entre os profissionais e usuários e estrutura organizacional dos serviços odontológicos Organização do acesso e desenvolvimento de ações Fatores que facilitam ou dificultam a realização de ações na APS Conhecimento sobre a rede de atenção à saúde bucal da PcD
Gestores dos CEO	Questionário	Tipo de CEO Oferta da especialidade “Odontologia para PNE” Recursos humanos para atendimento do PNE Condições de acessibilidade arquitetônica do CEO Número de cadeiras odontológicas existentes Disponibilidade de tecnologias assistivas para prover o atendimento adequado da PcD Organização e funcionamento dos serviços Aspectos relacionados à humanização dos serviços, à cordialidade e à qualidade das comunicações
Responsáveis pelo serviço de Odontologia da AH	Roteiro de entrevista semiestruturado	Características estruturais e de ambiência das unidades Disponibilidade de equipamentos, materiais, insumos, instrumentais Organização do processo de trabalho para atendimento às PcD
CDAH		
Acompanhantes de usuários atendidos na AH	Questionário	Condições de acessibilidade dos usuários ao hospital (custo, distância, tempo e meio de transporte utilizado para o deslocamento)

AH: atenção hospitalar; APS: atenção primária à saúde; CDAB: cirugião-dentista da atenção primária à saúde; CEO: Centros de Especialidades Odontológicas; PcD: pessoa com deficiência; PNE: paciente com necessidades especiais; RAS: rede de atenção à saúde; UBS: unidade básica de saúde.

Fonte: elaboração própria.

O questionário dos CDAB foi desenvolvido baseando-se em Macêdo et al. ^31^ e Rocha et al. ^32^, já o questionário dos gestores dos CEO e o *checklist* de materiais e equipamentos, no instrumento de avaliação externa do Programa Nacional de Melhoria do Acesso e da Qualidade dos Centros de Especialidades Odontológicas (PMAQ-CEO) ^33^, e nos compromissos pactuados para os CEO com adesão à RCPD ^17^. Os questionários online da plataforma REDCap (https://redcapbrasil.com.br/) ^34^ foram enviados por correio eletrônico ou aplicativo WhatsApp. Na AH, os acompanhantes de usuários agendados foram entrevistados no momento das visitas da pesquisadora às unidades.

A técnica de observação participante foi empregada em dois serviços hospitalares (H1 e H2) durante as visitas realizadas pela autora F.N.M.A., por um período de 12 horas em cada unidade. Foram utilizados um roteiro estruturado, fotografia e caderno de campo para documentar as atividades na sala de espera, nos ambulatórios e no centro cirúrgico, garantindo que as informações coletadas fossem abrangentes e representativas do contexto investigado. Foram verificados: estrutura física, equipamentos, recursos humanos, características do entorno do serviço e aspectos ligados à adequação (acolhimento, tempo de espera, fluxos de atendimento, organização e funcionamento dos serviços).

Além das fontes primárias, realizou-se um levantamento de informações de banco de dados do Sistema Nacional de Regulação (SISREG) fornecido pela Secretaria Municipal de Saúde do Rio de Janeiro (SMS-RJ) para caracterizar a demanda e a oferta dos serviços de Odontologia para PNE na rede pública municipal, após autorização da Coordenação do Complexo Regulador da SMS-RJ. Foram analisados: o tempo de espera para agendamento de consulta especializada na Odontologia para PNE, e as médias da distância (em km), do custo indireto (quantidade de meios de transporte) e do tempo de deslocamento (em minutos) das PcD aos CEO e ao AH em 2022. Para o cálculo dos indicadores, utilizou-se uma chave API do Google Maps (https://www.google.com/maps), obtida no Console de Desenvolvedor do Google, considerando-se os endereços do domicílio do usuário e da unidade executante (CEO ou hospital).

Com base nas informações levantadas, os critérios/indicadores foram comparados a padrões determinados, cujos valores numéricos de referência foram retirados de documentos institucionais, legislação ou da literatura científica. Os valores dos indicadores foram normalizados, em uma escala de 0 a 1, considerando-se o valor de pertinência 1 como a melhor situação e o valor 0 como a pior situação.

O somatório dos pontos atribuídos a cada indicador resultou na pontuação de cada dimensão. Com o objetivo de equilíbrio entre as dimensões do acesso, foi realizada a classificação de relevância variando entre R (relevante), RR (muito relevante) e RRR (extremamente relevante), como previsto pela metodologia de implantação. Levando-se em conta a pontuação final das dimensões, foram definidos quatro estratos para julgamento do acesso da PcD aos serviços de saúde bucal, baseados em Alves et al. ^35^: acesso satisfatório ou adequado (75% a 100%; 201,7-269 pontos); acesso parcial ou parcialmente adequado (50% a > 75%; 134,5-201,6 pontos); acesso incipiente (25% a > 50%; 67,2-134,4 pontos); e acesso desfavorável ou crítico (1% a > 25%; 0-67,1 ponto). Esses estratos correspondem ao grau de implantação da PNSDP referente à atenção odontológica à PcD.

A pesquisa foi aprovada pelos comitês de ética em pesquisa da Escola Nacional de Saúde Pública Sergio Arouca, Fundação Oswaldo Cruz (ENSP/FIOCRUZ; CAAE 57966522.3.0000.5240), e da SMS-RJ (CAAE 57966522.3.3001.5279).

## Resultados

A soma da pontuação atribuída a cada dimensão e suas respectivas subdimensões resultou na pontuação final de 126,15 (Tabela 1). Considerando-se os estratos para julgamento, esse valor correspondeu a 46,89%, que classificou o acesso da PcD aos serviços de saúde bucal no município como “incipiente”.

**Tabela 1:** Matriz síntese dos valores obtidos dos indicadores, de acordo com as dimensões e subdimensões do acesso da pessoa com deficiência (PcD) aos serviços de saúde bucal da rede pública do Município do Rio de Janeiro, Brasil, 2022.

Dimensões/Subdimensões	Componentes	Valores de referência			Valores observados		
		Indicadores	Relevância	Pontuação máxima final com relevância	Pontuação final	Pontuação final com relevância	Pontuação final da dimensão
Acessibilidade							
Acessibilidade arquitetônica	Condições de acessibilidade arquitetônica das unidades de saúde	41	R	41	20,68	20,68	42,08
Acessibilidade geográfica	Condições de acessibilidade geográfica às unidades de saúde	19	RR	38	10,70	21,40	
Adequação							
Qualificação profissional	Qualidade técnica	8	RR	16	2,01	4,02	40,14
Organização da oferta de cuidados	Organização e integralidade do cuidado	7	RR	78	4,77	9,54	
	Processo de trabalho	17			9,33	18,66	
	Construção compartilhada do cuidado	15			3,96	7,92	
Disponibilidade							
Estrutura operacional da RAS	Apoio e logística	8	RR	38	4,41	8,82	43,93
	Oferta de serviços de atenção à saúde bucal	11			5,51	11,02	
Recursos	Recursos humanos	9	RR	18	2,7	5,4	
	Recursos materiais	28	R (gerais)	22	18,27	18,27	
			RRR (específicos)	6	0,14	0,42	
Valores totais		163	-	269	85,46		126,15

Fonte: elaboração própria.

**Figura 2:**
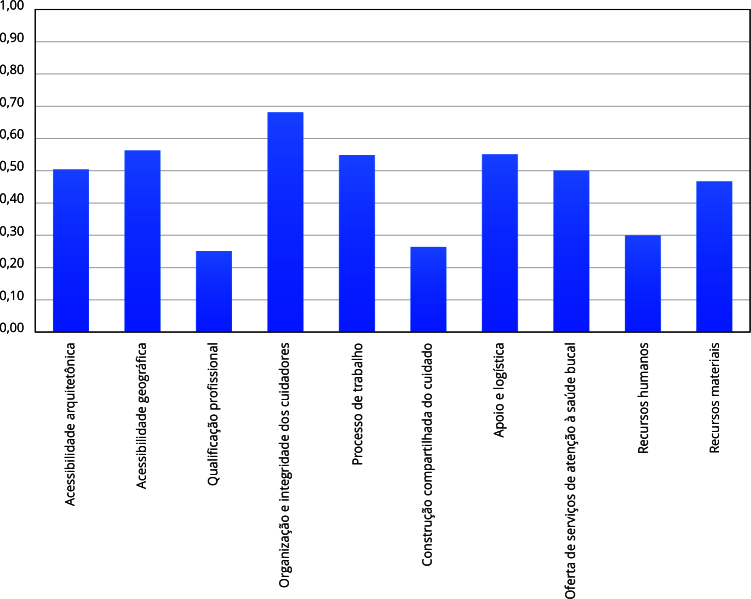
Grau de implantação da atenção à saúde bucal à pessoa com deficiência nos serviços da rede pública do Município do Rio de Janeiro, Brasil, segundo as dimensões do acesso, acessibilidade, adequação e disponibilidade, e suas respectivas dimensões, 2022.

Baseando-se nos resultados da matriz síntese (Tabela 1) foi realizada a comparação entre os valores obtidos em cada subdimensão do acesso por meio da Figura 2. Os valores observados e de referência foram normalizados com o objetivo de compatibilizá-los e de que passassem a ter o mesmo intervalo, variando em uma escala de 0 a 1.

Fonte: elaboração própria.

Os pontos mais críticos no processo de implantação da PNSPD referente à atenção à saúde bucal no município estavam relacionados às subdimensões: qualificação profissional, construção compartilhada do cuidado, e recursos humanos e materiais. A acessibilidade arquitetônica e a oferta de serviços também se destacaram de forma negativa. A seguir serão apresentados os resultados referentes a cada dimensão.

### Acessibilidade

Para avaliar a acessibilidade, foram considerados aspectos que pudessem limitar ou impedir o acesso, a liberdade de movimento e a circulação com segurança das PcD nas unidades de saúde, como condições de deslocamento do usuário até a UBS (unidades básicas de saúde) e a distribuição geográfica dos CEO e hospitais de referência no município.

Verificou-se que, de forma geral, as unidades contavam com rampas de acesso, portas e corredores largos para a passagem de cadeira de rodas, mas faltaram piso tátil e sinalização em Braille, adequação das salas de espera e de banheiros, e estacionamentos destinados para PcD.

Na APS e na AE, a acessibilidade arquitetônica foi classificada como “muito ruim”. Sessenta e cinco por cento dos CD consideraram as instalações da UBS acessíveis ao PcD, quase 40% apontaram a inexistência de banheiros adaptados, 80% a ausência de sinalização em Braille, de vagas de estacionamento e sala de espera adequada em metade das unidades. Setenta e sete vírgula oito por cento dos gestores do CEO responderam que não havia cadeira de rodas em condições de uso, banheiro adaptado e sala de espera adequada. Oitenta e três vírgula três por cento apontaram que os CEO não contavam com vaga de estacionamento para PcD, informação em Braille ou piso tátil. Muitos CEO tinham estrutura antiga: “*precisaria criar um elevador por fora ou mesmo uma plataforma para cadeirante*” (GC3). Apesar disso, 61,1% deles consideraram as instalações do CEO acessíveis.

Já na AH, a acessibilidade arquitetônica foi classificada como “ruim”. As unidades apresentavam estrutura antiga, necessitando adequação, renovação do mobiliário e manutenção predial. O centro cirúrgico do H2 apresentava corredores e portas largas para acesso às salas operatórias. Os atendimentos sob anestesia do H1 eram realizados em sala pequena e com portas estreitas que dificultavam a passagem da maca e a transferência do paciente à cadeira odontológica. Apesar dos usuários terem acesso ao estacionamento do H1, não havia vagas para PcD no H2.

Nas duas unidades não havia piso tátil nem informações em Braille, mas os ambulatórios odontológicos permitiam mobilidade e acesso de cadeiras de rodas. No H1, havia sanitários no local de espera com rampa de acesso junto à soleira e apenas uma barra de apoio ao assento sanitário. No H2, os sanitários do ambulatório apresentavam desnível junto à soleira, portas estreitas, ausência de área para a transferência para bacia sanitária e barra de apoio, ou área de manobra/circulação. No H2, todos os acompanhantes dos usuários consideraram fácil, no H1, 40% deles classificaram a acessibilidade arquitetônica como moderada. A1 comentou: “*como o filho andava, a acessibilidade era fácil, mas para um cadeirante não*” e A9 referiu que o filho não tinha “*coordenação*”. A8 considerou a acessibilidade moderada devido à “*baixa visão*” da filha e que se não segurasse sua mão ela tropeçaria.

Quanto à acessibilidade geográfica, os usuários percorreram, em média 6,41km, levando 32,31 minutos no trajeto e utilizando 1,12 meio de transporte para se deslocar ao CEO em 2022, classificando a distância como “muito boa”, o tempo como “bom” e o custo “bom”. Pela média desses três indicadores, o deslocamento da PcD ao CEO foi considerado “bom”. Metade dos gestores dos CEO disse que a localização do centro odontológico era conveniente para a PcD utilizar transporte público. Para o deslocamento ao hospital, os usuários percorreram em média 26,77km em 82,65 minutos, utilizando 2,05 meios de transporte no trajeto. A distância foi classificada como “regular”, o tempo como “ruim” e o custo “muito ruim”. A média dos indicadores classificou o deslocamento da PcD à AH como “ruim”. Os usuários das AP 5.2 e 5.3 percorreram a maior distância, gastaram o maior tempo e usaram mais meios de transporte para o deslocamento aos CEO e hospitais, enquanto os da AP 2.1 chegavam mais rápido aos CEO. Para o atendimento na AH, os usuários da AP 1.0 percorreram a menor distância, e os da AP 3.2 gastaram o menor tempo e utilizaram a menor quantidade de meios de transporte.

Quanto à localização das unidades, havia maior concentração de CEO nas AP 1.0 e 3.1, sendo que na AP 5.3 o CEO não oferta atendimento na especialidade Odontologia para PNE e nas AP 3.3 e na 5.1 apenas um ofertava este serviço. Os hospitais de referência estavam localizados nas AP 1 e 2.2, e os usuários das AP 4.0, 5.1, 5.2 e 5.3 percorreram a maior distância com maior tempo e custo.

O deslocamento da PcD/PNE até as UBS foi classificado pelos CDAB como “moderado”. Para 85,6%, os domicílios tinham condições precárias de acessibilidade (diversos andares, com corredores estreitos e escadas) e havia terreno acidentado/íngreme (75,5%) e sem pavimentação (74,8%) no trajeto domicílio/UBS. Cinquenta por cento dos CD disseram haver barricadas nos territórios e 60% referiram episódios de confrontos armados; A9 relatou: “*Uber não quer ir por que é comunidade, cancela pela violência*”.

O deslocamento da PcD/PNE até as unidades de atenção odontológica especializada foi considerado como “difícil” pelos CDAB. A16 declarou: “*ando da casa ao ponto de trem e tem acessibilidade péssima. Do trem até a unidade vou a pé. A rua é inclinada e as calçadas muito esburacadas. É um sacrifício. Não tem ônibus que deixe na porta do hospital*”.

Para chegar à unidade de AH, os acompanhantes utilizam: ônibus (38,1%), trem (9,5%), metrô (9,5%), táxi/transporte de aplicativo (38,1%), carro próprio (19%) e van (9,5%). Segundo A21, “*se fosse que vir por transporte público não saberia, seria difícil. Teria que pegar trem, depois ônibus e vir a pé*” e A3 “*pegava 2 ônibus, trem e tinha que andar cerca de 10 minutos, atravessando uma passarela com rampa*”. “*Os ônibus são precários... o elevador não funciona, o cinto de segurança para cadeirante no trem é quebrado... Para subir na estação tem elevador, mas para descer da catraca até o trem só tem escada e são os seguranças que ajudam*” (A16). A falta de transporte eletivo em saúde foi destacada: “*Transporte é suporte que o Estado não dá. A locomoção dele é difícil para subir no ônibus... é pesado e se agita do nada*” (A9). Muitos optavam pelo transporte por aplicativo: “*Como vim de Uber achei fácil chegar, mas se tivesse de transporte público, seria difícil porque teria que pegar 2 ônibus*” (A11). O transporte por aplicativo implicava o custo adicional às famílias.

### Adequação

Nesta dimensão, foi avaliado como os serviços se organizavam para receber os usuários e sua adaptação à organização. Quanto à organização da oferta de cuidados, analisou-se os critérios e fluxos da RAS (existência de protocolo para encaminhamento à especialidade PNE e o conhecimento dos CD sobre critérios e fluxos da rede estabelecidos neste protocolo); o processo de trabalho da APS, AE e AH (acolhimento, acompanhamento, atendimento e referência de casos que demandam cuidados especializados em Odontologia à AE e/ou AH). Indicadores da articulação e integração profissional para a construção compartilhada do cuidado consideraram a interdisciplinaridade e o trabalho em equipe. Os indicadores da qualificação profissional foram: formação profissional dos CD dos CEO e da AH em Odontologia para PNE; realização da disciplina Odontologia para PNE pelo CDAB na graduação; realização de cursos/ treinamentos para atendimento da PcD/PNE; e sensação de segurança do CDAB para atender PcD/PNE.

Quanto à qualificação profissional, 64% dos CDAB referiram não ter cursado disciplina na graduação sobre o tema e 36,7% não se sentiam seguros para realizar o atendimento da PcD/PNE. Sentiam necessidade de se atualizar 93,5%, 64% disseram não ter oferta de cursos no tema PcD, e 82% nunca participaram de treinamento. Houve divergência entre profissionais e gestão quanto às atividades programadas na agenda de Educação Permanente.

Na AE, os gestores de CEO responderam que 32% dos CD possuíam especialização/aperfeiçoamento na área. Apenas 38,9% dos CD e 5,6% dos auxiliares (ASB) e técnicos de saúde bucal (TSB) haviam participado de treinamentos nos últimos cinco anos. De fato, o CDAE considerou que ASB e TSB não têm esse perfil voltado para Odontologia de PNE e que a formação destes profissionais é um problema para o atendimento dos pacientes.

Na AH, os CD apresentaram como demanda realizar treinamento específico em sedação, especialmente a inalatória com óxido nitroso. Para o CD da AE “*é de suma importância ter sedação nos CEO... ter um especialista que consiga proporcionar ao paciente esse tratamento*”, mas a utilização do óxido nitroso apresenta a necessidade da máscara nasal e exige a “*mínima colaboração do paciente, reduzindo a indicação na Odontologia para Pacientes Especiais... o uso da sedação medicamentosa a nível ambulatorial ou indicação de tratamento sob anestesia geral são as opções mais indicadas*” (CDAE).

Verificou-se que o município tinha o protocolo da especialidade de Odontologia para PNE e 99% dos CDAB afirmaram conhecer os critérios e fluxos para encaminhar o usuário aos serviços de AE/AH. Os CDAH demonstraram insatisfação quanto aos encaminhamentos realizados pelos CDAB, já que os procedimentos que realizavam no hospital poderiam ser realizados na APS.

O processo de trabalho para atenção à saúde bucal das PcD/PNE foi classificado como “ruim” nos três níveis de atenção. Na APS, apesar de 61,6% dos CD conhecerem as PcD residentes na área de atuação, somente 10,1% sabiam o perfil das PcD (quantidade, tipos de deficiência, demanda de cuidados em Odontologia). Na AE, 94% dos CEO não disponibilizavam de tempo exclusivo para o atendimento do PNE.

Na AH não havia atendimento odontológico em caráter de urgência. No H1 eram realizados somente dois atendimentos sob anestesia geral em um turno semanal, e no H2 eram quatro em dois turnos, tornando o tempo de espera longo (mais de 1 ano no H1 e de 3 meses no H2) e não havia critérios definidos para a indicação de atendimento sob anestesia geral nem para a ordenação da fila de espera.

A construção compartilhada do cuidado para atenção à saúde bucal da PcD/PNE foi considerada péssima. Na APS, havia pouco suporte aos CD pelos profissionais das equipes multiprofissionais na APS (eMulti) e do CEO. Na AE, não eram desenvolvidas ações de matriciamento pelos profissionais do CEO junto à APS e, na AH, a relação com os demais profissionais da AH para a construção de plano terapêutico do paciente era pontual e frágil.

### Disponibilidade

Nesta dimensão, verificou-se o volume e os tipos de recursos e serviços existentes para a atenção à saúde bucal da PcD relacionados à “estrutura operacional da RAS” (sistemas de apoio e logístico como registro eletrônico, acesso regulado, transporte em saúde e manutenção de equipamentos dos serviços de atenção à saúde bucal) e à “oferta de serviços de atenção à saúde bucal” (cobertura de saúde bucal na APS e populacional dos CEO, oferta da especialidade “Odontologia para PNE” nos CEO, adesão dos CEO à RCPD e existência de centro cirúrgico equipado para atendimento odontológico da PcD nos hospitais). Foi analisada a disponibilidade de recursos humanos (quantidade de CDAB em relação à população do município, de CD atuantes na especialidade PNE, de ASB/TSB nos CEO, e de CD, ASB, TSB e anestesistas nas unidades de AH). Verificou-se se havia insumos instrumentais e equipamentos gerais e específicos para o atendimento odontológico da PcD/PNE em cada nível de atenção.

O componente “Apoio e logística” foi classificado como “regular”. O sistema de prontuário eletrônico havia sido implantado amplamente na APS, mas parcialmente na AE. Na AH, era utilizado prontuário físico. Quanto ao sistema de transporte sanitário eletivo, 53,2% dos CDAB informaram não dispor de veículo adaptado para o acesso da PcD/PNE aos serviços de saúde bucal especializados.

Quanto à abrangência e distribuição dos serviços, verificou-se que a cobertura de saúde bucal na APS no município era de 25% em agosto de 2023. Em 2022, nem todos os CEO ofereciam atendimento de “Odontologia para PNE”, especialidade mínima exigida, e apenas 16,7% deles tinham adesão à RCPD. Na AH, não havia oferta de atendimento odontológico ambulatorial/cirúrgico para PcD/PNE 40 horas/semanais.

As solicitações para consulta de “Odontologia para PNE” foram predominantemente para pacientes adultos do sexo masculino, sendo a maior parte de retorno, agendadas pelas próprias unidades executantes (CEO e hospital). Em 2022, o tempo de espera para 1ª consulta no CEO foi, em média, de 26 dias e na AH de 52 dias. A disponibilidade de vagas para atendimento odontológico do PNE no município foi considerada suficiente em relação à demanda de solicitações realizadas no SISREG pela gestão. Os gestores de CEO realmente informaram que as vagas para essa especialidade vinham sendo parcialmente (55,6%) ou raramente ocupadas (16,7%).

A disponibilidade de recursos humanos para a atenção à saúde bucal da PcD/PNE foi considerada péssima. Na APS, a quantidade necessária de CD em relação à população coberta pela ESF no município foi considerada insuficiente, com as eSB responsáveis por três ou mais equipes de saúde da família. Na AE e AH, a quantidade de TSB e ASB foi considerada insuficiente pelos gestores dos CEO (72,2% e 94,4%, respectivamente). Na AH, observou-se que técnicos de enfermagem davam apoio aos profissionais de saúde bucal. Foi apontada a insuficiência de CD: “*Precisamos de mais dentistas, mas eles foram se aposentando e não veio mais ninguém*” (GL20). Outra questão foi a pouca disponibilidade de anestesistas para atendimento do PNE na AH. O tipo de contrato e vínculo trabalhista dos profissionais foi apontado como desafio: “*A forma de contratação é sempre muito difícil, a gente não consegue evoluir porque você não consegue ter servidor*” (GC4).

A disponibilidade de recursos materiais foi classificada como “regular”. Na APS, a maioria dos CD respondeu que sempre ou frequentemente dispunha de materiais e insumos gerais para o atendimento das PcD/PNE. Nos CEO, gestores informaram não dispor de recursos específicos como aparelho para analgesia inalatória e sedação consciente por óxido nitroso, e de materiais para auxiliar no posicionamento e estabilização protetora do paciente na cadeira odontológica. Em três CEO havia aparelho para analgesia, mas em apenas um era utilizado: “*O aparelho de sedação está na caixa há mais de 10 anos porque não foi feita capacitação, não sei se funciona mais*” (GL8). Na AH, também faltavam insumos, materiais e equipamentos específicos para o atendimento da PcD/PNE. No centro cirúrgico do H2 não havia cadeira odontológica. Não havia aparelho de radiografia dentária nas unidades hospitalares.

## Discussão

Com base nos indicadores da matriz de análise e julgamento, e do Modelo Lógico de Atenção à Saúde na RCPD ^24^, foi realizada a avaliação da atenção à saúde bucal às PcD no Município do Rio de Janeiro. Os pontos mais críticos no processo de implantação foram: qualificação profissional, construção compartilhada do cuidado, e recursos humanos e materiais, seguido da acessibilidade arquitetônica e oferta de serviços.

Quanto à acessibilidade, as principais barreiras foram: longa distância dos hospitais a determinadas regiões da cidade, baixa qualidade do transporte público (barreiras físicas e atitudinais), pouca integração entre modais de transporte, violência urbana e inadequação das vias públicas.

No Município do Rio de Janeiro, as especificações técnicas e os critérios para a acessibilidade não eram cumpridos integralmente, especialmente nos hospitais inaugurados na primeira metade do século XX e CEO que funcionavam dentro de antigas policlínicas. No estudo de Condessa et al. ^36^, realizado nas cinco macrorregiões brasileiras, apenas 30,4% dos CEO apresentavam acessibilidade física adequada. Outros estudos apontam que o descumprimento das regras de acessibilidade às UBS ^37^ e a falta de estrutura adequada para as PcD ^32^ ^,^ ^38^ geram barreiras ao cuidado ^39^, sendo fundamental o planejamento de construções ou reformas de acordo com as normas de acessibilidade ^32^ e a implantação de medidas em diversos aspectos para promovê-la ^37^ ^,^ ^40^.

Quanto à acessibilidade geográfica, a distribuição territorial desigual dos CEO e hospitais em relação às AP é um dificultador para o deslocamento dos usuários às unidades de atendimento especializado. Havia grande discrepância entre os valores referentes à distância e tempo de deslocamento de acordo com as AP solicitantes, com a necessidade de investimento diferenciado em acessibilidade na AP ^5^. Dificuldades de deslocamento até os serviços de saúde foram identificadas pela maioria dos dentistas, comprometendo a acessibilidade ^32^. Gomes et al. ^41^ mostraram que as longas distâncias para o local da consulta/exame também foram reconhecidas pelos médicos que atuam na APS do Rio de Janeiro, gerando inclusive absenteísmo em função de limitações dos usuários (inclusive financeiras) para se locomover na cidade.

Souza Filho et al. ^42^ trazem que as condições de acessibilidade do transporte público impedem acesso e mobilidade plena e autônoma das PcD, tendo seu direito cerceado, sobretudo aquelas que habitam em regiões mais distantes, onde há escassez de serviços públicos básicos. Consequentemente, a falta de transporte sanitário eletivo gerava custos financeiros adicionais e diretos às famílias das PcD, que optavam pelo transporte por aplicativo ou solicitavam apoio a vizinhos e amigos.

Em um município onde grande parte da população reside em áreas vulnerabilizadas e em contexto de pobreza e violência urbana, a falta de acessibilidade e as condições precárias dos domicílios são mais uma barreira, exigindo programas de urbanização de favelas ^43^ e tornando o acesso universal à saúde ainda mais complexo e difícil ^44^.

Na dimensão adequação, verificou-se a sensação de insegurança do CDAB para realizar o atendimento da PcD/PNE atrelada à insuficiente oferta de oportunidades para qualificação profissional. Investimento e estímulo aos profissionais da rede para a qualificação da atenção à saúde bucal das PcD é necessário porque, como mostrado por Da Rosa et al. ^45^, grande parte dos CD não se sentiam seguros e preparados para realizar o atendimento das PcD na APS. A baixa frequência de ações de matriciamento desenvolvidas pelos profissionais do CEO juntos às eSB da APS e a fraca relação interdisciplinar entre os profissionais de saúde bucal com as demais categorias e equipes são barreiras para a construção compartilhada do cuidado.

Quanto à organização da oferta de cuidados, apesar de o município ter o protocolo da especialidade de Odontologia para PNE, havia descaracterização das competências e atribuições dos níveis de atenção à saúde bucal das PcD/PNE. Além disso, havia desajustes entre o que era estabelecido pelo protocolo como critério para o encaminhamento e o que ocorria nos fluxos entre os serviços de APS e AE/AH, o que poderia ser justificado pela insegurança profissional para realizar este atendimento, referenciando casos que poderiam ser solucionados na APS ^36^ ^,^ ^46^ ou por dificuldade de acompanhamento e prestação do cuidado pelos profissionais desta atenção de saúde ^47^.

Dentre as barreiras ligadas à oferta de serviços, verificou-se baixa cobertura de saúde bucal na APS, baixa adesão dos CEO à RCPD, a não oferta da especialidade mínima de “Odontologia para PNE” em todos os CEO, a indisponibilidade de 40 horas semanais para atendimento ambulatorial da PcD nos CEO e na AH, e oferta limitada de atendimento odontológico sob anestesia geral.

Pelo fato de a cobertura de saúde bucal da APS no Município do Rio de Janeiro em 2022 estar abaixo da cobertura nacional estabelecida (40,4%) ^48^, a sobrecarga de funções desempenhadas pelos CD poderia justificar as fragilidades de identificação e levantamento de necessidades de saúde bucal das PcD, pois a população de referência é maior do que a capacidade da equipe. Como apontado por Carneiro et al. ^49^, a baixa cobertura de saúde bucal na APS traz dificuldades para a coordenação do cuidado pela sobrecarga dos demais serviços e desorganização dos fluxos de acesso, resultando em descontinuidade e fragmentação do cuidado. Consequentemente, a descaracterização da APS como coordenadora do cuidado impactava o próprio ordenamento de fluxos na rede de atenção à saúde, pois, segundo Santos et al. ^50^, se refletia no aumento do número de casos referenciados para o atendimento odontológico com o uso de anestesia geral.

De forma semelhante aos resultados deste estudo, verificou-se insatisfação quanto aos encaminhamentos realizados pelos CDAB para a AH ^47^, sinalizando haver poucas iniciativas de colaboração técnica com outros pontos de atenção para a qualificação da rede de serviços. Carneiro et al. ^51^ também mostraram que, nas unidades das regiões de São José do Rio Preto (São Paulo) e de Salvador (Bahia), a comunicação e a interação entre os profissionais de unidades de saúde operavam em uma lógica isolada e fragmentada de prestação de serviços básicos e especializados, não estruturando recursos para as ações de cuidado. Isso mostra que a ausência de comunicação entre serviços e profissionais atua como inibidor do acesso ^39^.

Apesar de constar no protocolo municipal que após a conclusão do tratamento hospitalar haja contrarreferência do usuário à unidade de origem (solicitante), o CDAH muitas vezes se responsabilizava pelo cuidado do usuário realizando procedimentos de APS em consultas de revisão ambulatorial. Cabe dizer que, enquanto os hospitais estavam sobrecarregados, as vagas para a especialidade em questão estavam sendo parcialmente ocupadas nos CEO. Assim, o papel do CEO na rede precisa ser rediscutido, incluindo a implantação de ações de matriciamento junto às equipes da APS e de sedação medicamentosa em nível ambulatorial mediante treinamento profissional.

Assim, a fraca articulação e integração entre os diferentes pontos de atenção, evidenciada pela ausência do sistema de prontuário eletrônico na AE e na AH, pela fragilidade no sistema de referência e contrarreferência e de apoio matricial, implicavam nas dificuldades para o alcance da oferta do cuidado integral à PcD.

De forma semelhante ao Município do Rio de Janeiro, Condessa et al. ^36^ salientaram que não obstante estar prevista como atendimento obrigatório a ser prestado pelo CEO, 10,2% deles não ofereciam atendimento nessa especialidade. Considerando que 51,9% dos CEO no Brasil (615 de 1.186) tinham adesão à RCPD em 2023 ^52^, no Município do Rio de Janeiro apenas 16,7% deles haviam sido homologados.

Ao contrário dos resultados encontrados neste estudo, Condessa et al. ^36^ mostraram que o tempo de espera para atendimento da PNE nos CEO nas cinco macrorregiões no Brasil era pequeno, com mais da metade dos usuários atendidos, em média, uma semana depois de serem encaminhados. Já a fila de espera para atendimento odontológico sob sedação/anestesia geral indicou que a oferta do serviço era insuficiente frente à demanda das PcD/PNE. É necessário compreender os fatores relacionados à oferta de serviços (estrutura e processo) e características da demanda na gestão da fila de espera para cirurgias eletivas ^53^. Conhecer o perfil demográfico e assistencial dos usuários, utilizar parâmetros referenciais para o planejamento e a programação das ações e serviços de saúde bucal, assim como aprimorar o processo de avaliação com critérios clínicos adequados é fundamental para o ordenamento e a indicação de tratamento odontológico sob anestesia geral. Devido ao longo tempo transcorrido entre a avaliação odontológica inicial e o atendimento no centro cirúrgico, há risco de agravamento do estado de saúde do paciente ^53^ ^,^ ^54^.

É importante destacar que o tempo real de espera para atendimento odontológico da PcD/PNE sob anestesia geral não aparecia no sistema de regulação, mas nas filas “invisíveis” que existiam dentro das próprias unidades hospitalares. O agendamento da primeira consulta por meio do SISREG não proporcionava maior agilidade de atendimento e mostrava que o longo tempo de espera para consultas especializadas é uma barreira ao acesso a cuidados integrais à saúde no SUS ^55^. Diante da grande demanda de tratamento cirúrgico-restaurador nos pacientes especiais e da baixa cobertura odontológica hospitalar da população com deficiência no Brasil ^56^, há necessidade de ampliar a oferta do serviço odontológico hospitalar ^56^ ^,^ ^57^. Outra questão é a incipiente formação desse profissional nessa área ^50^.

Quanto à disponibilidade de recursos humanos, considerou-se como dificultadores: a desproporção entre o número de eSB da APS e equipes de saúde da família; a quantidade insuficiente de ASB e TSB nos CEO e nos hospitais de referência; além da quantidade insuficiente de CDAH e de médicos anestesistas para a atenção à saúde bucal das PcD/PNE. Não houve avanços ao longo do processo de implantação da PNSPD no município. Pelo contrário, houve flexibilização e precarização do trabalho, colocando em questão a sustentabilidade dos serviços baseados no modelo das Organizações Sociais em Saúde (OSS) ^58^. O provimento e a fixação de profissionais, especialmente em áreas conflagradas pela violência, se traduzem em dificuldade para a fixação dos mesmos. Quanto à disponibilidade de anestesistas, apesar de a Região Sudeste concentrar a maior parte dos especialistas nesta área, um grande número atua no setor privado de saúde ^59^. Em relação aos recursos materiais, a falta de insumos e equipamentos específicos foi o principal fator dificultador para atendimento odontológico da PcD.

Por fim, a inadequada gestão e governança das políticas públicas locais, incluindo a falta de recursos, afeta o acesso aos serviços de saúde, inclusive das PcD. Verificou-se que os gestores entrevistados já não ocupavam o cargo quando a pesquisa foi finalizada. Isso demonstra a alta rotatividade dos gestores em função da relação com os processos partidários e eleitorais e a baixa capacidade gerencial, que está distante das necessidades e complexidades do setor, gerando descontinuidade, permanentes recomeços e desmotivação dos profissionais ^60^.

Como apresentado por Carneiro et al. ^49^, diferenças na capacidade de produção dos serviços entre as regiões brasileiras são compatíveis com as discrepâncias na estrutura disponível para a assistência à PcD e à população em geral. Isso mostra que ainda que o governo federal exerça papel regulador e defina incentivos, o processo de regionalização é afetado pelo contexto, comportamento, capacidade técnica e opções políticas dos atores regionais que são autônomos para a decisão e a organização dos seus respectivos territórios.

## Considerações finais

O acesso da PcD aos serviços de saúde bucal da rede pública do Município do Rio de Janeiro ainda é “incipiente”, havendo mais barreiras do que fatores facilitadores. A implantação da política vem ocorrendo de forma paulatina com fragilidades na integração e cooperação entre os atores estratégicos na governança da rede, não se considerando de forma consistente as necessidades de saúde bucal das PcD, a capacidade instalada ambulatorial e hospitalar nem a organização dos pontos de atenção à saúde a este público.

Por se tratar de uma pesquisa avaliativa conduzida durante a implantação da PNSPD para apoiar o seu desenvolvimento, são apresentadas recomendações direcionadas à gestão central (macrogestão), regional (mesogestão) e local (microgestão), de acordo com os níveis de influência dos atores envolvidos na governança da rede.

À macrogestão, sugere-se verificar a existência de critérios básicos de promoção da acessibilidade nas unidades de saúde, articulação de ações para investimento no transporte público da cidade e oferta de transporte sanitário eletivo para garantir o acesso das PcD aos serviços de saúde.

A pesquisa aponta a necessidade de requalificação das unidades de saúde, de eliminação de barreiras arquitetônicas e organizacionais para a promoção do acesso com equidade.

Recomenda-se a parceria com instituições de ensino e universidades para a qualificação dos profissionais da rede. Para a organização e a integralidade do cuidado à PcD, é preciso constante atualização e disseminação dos critérios e fluxos estabelecidos no protocolo de atendimento odontológico. À mesogestão, recomenda-se promover integração entre os profissionais das eMulti e dos CEO com a APS e implantar ações de matriciamento. Para a integração e o compartilhamento das informações entre os níveis de atenção da rede, deve-se implantar e integrar o sistema de prontuário eletrônico.

É fundamental que na microgestão os CD conheçam a identidade e a real necessidade de cuidados em saúde bucal das PcD, e que a gestão central busque a ampliação da cobertura de saúde bucal. Recomenda-se ainda a regulação centralizada das vagas para atendimento odontológico sob anestesia geral, com a classificação de risco e o ordenamento da fila de espera baseados em critérios bem definidos e oferta ampliada para hospitais gerais, além dos especializados. Aponta-se a possibilidade de implantação da sedação medicamentosa em nível ambulatorial no CEO mediante treinamento profissional.

Para acompanhar a implantação da atenção à saúde bucal à PcD, recomenda-se à macro e à mesogestão adotar um programa contínuo de autoavaliação das ações desenvolvidas, de acordo com as especificidades de cada nível de atenção. Ademais, é crucial que haja participação e controle social, qualificação da gestão e corresponsabilização entre os atores que a integram a RCPD para a implantação da PNSPD, estimulando o protagonismo e garantindo a inclusão social das PcD, pois a invisibilidade dessas pessoas se perpetua, apesar da instituição de normas federais.

A superação dos desafios para a oferta de cuidados às PcD/PNE nos serviços de saúde depende essencialmente da coordenação entre profissionais de saúde, usuários, cuidadores e da infraestrutura global. Os diversos aspectos que se relacionam ao acesso têm um caráter multifacetado, o que requer abordagem em múltiplas perspectivas, sejam elas biológicas, sociais, físicas e financeiras, pois nenhuma delas contribui única e exclusivamente para a atenção à saúde desse público.

Como limitações, a pesquisa não alcançou a taxa de resposta desejada dos CDAB em determinadas AP. Atribuiu-se à dificuldade de divulgação da pesquisa e envio do *link* dos questionários online a todos os profissionais, já que a gestão disponibilizou somente os e-mails das equipes de saúde bucal. Outra limitação é que o questionário dos usuários não considerou questões relacionadas à participação efetiva das PcD na defesa de seus interesses e judicialização para acesso à rede. Quanto ao banco do SISREG, inconsistências de dados decorrentes de erros de preenchimento e não padronização das informações levaram à necessidade de limpeza ostensiva do banco. Para superar as limitações relacionadas à parte quantitativa, considerou-se a complementariedade do ponto de vista qualitativo, pois as informações foram interpretadas por diferentes abordagens metodológicas.

O recorte temporal e espacial da avaliação em um município de grande porte da Região Sudeste do Brasil foi outra limitação. Assim, há necessidade de avaliação ao longo da implantação da PNSPD neste e em demais contextos. Ademais, a pesquisa tomou como foco a oferta de serviços às PcD com necessidades de cuidados especializados em Odontologia, sendo imprescindível uma avaliação mais global do acesso dessas pessoas.

Os resultados deste estudo evidenciam problemas estruturais comuns no SUS e em outros sistemas de saúde do mundo, o que pode ajudar a superar barreiras e a formular políticas públicas mais eficazes e alinhadas às necessidades reais dos serviços e das PcD. Apesar das limitações, este estudo é uma contribuição importante para que gestores, profissionais e avaliadores possam acompanhar as ações desenvolvidas por meio de critérios e indicadores da matriz de análise e julgamento, buscando o cuidado integral à saúde das PcD.

## Data Availability

As fontes de informação utilizadas no estudo estão indicadas no corpo do artigo.
